# A Real-Time Sensing System for Monitoring Neural Network Degeneration in an Alzheimer’s Disease-on-a-Chip Model

**DOI:** 10.3390/pharmaceutics14051022

**Published:** 2022-05-09

**Authors:** Nien-Che Liu, Chu-Chun Liang, Yi-Chen Ethan Li, I-Chi Lee

**Affiliations:** 1 Department of Biomedical Engineering and Environmental Sciences, National Tsing Hua University, Hsinchu 300044, Taiwan; h0a9n1k8@gmail.com (N.-C.L.); vangie.liang110@gmail.com (C.-C.L.); 2 Department of Chemical Engineering, Feng Chia University, Taichung 407102, Taiwan; yicli@fcu.edu.tw

**Keywords:** neural stem cells (NSCs), neural network formation, biochip, Alzheimer’s disease-on-a-chip model, β-amyloid (Aβ), real-time impedance analysis

## Abstract

Stem cell-based in vitro models may provide potential therapeutic strategies and allow drug screening for neurodegenerative diseases, including Alzheimer’s disease (AD). Herein, we develop a neural stem cell (NSC) spheroid-based biochip that is characterized by a brain-like structure, well-defined neural differentiation, and neural network formation, representing a brain-on-a-chip. This system consisted of microelectrode arrays with a multichannel platform and allowed the real-time monitoring of network formation and degeneration by impedance analysis. The parameters of this platform for the real-time tracking of network development and organization were established based on our previous study. Subsequently, β-amyloid (Aβ) was added into the brain-on-a-chip system to generate an AD-on-a-chip model, and toxic effects on neurons and the degeneration of synapses were observed. The AD-on-a-chip model may help us to investigate the neurotoxicity of Aβ on neurons and neural networks in real time. Aβ causes neural damage and accumulates around neurites or inside neurospheroids, as observed by immunostaining and scanning electron microscopy (SEM). After incubation with Aβ, reactive oxygen species (ROS) increased, synapse function decreased, and the neurotransmitter-acetylcholine (ACh) concentration decreased were observed. Most importantly, the real-time analysis system monitored the impedance value variation in the system with Aβ incubation, providing consecutive network disconnection data that are consistent with biological data. This platform provides simple, real-time, and convenient sensing to monitor the network microenvironment. The proposed AD-on-a-chip model enhances the understanding of neurological pathology, and the development of this model provides an alternative for the study of drug discovery and cell–protein interactions in the brain.

## 1. Introduction

Neurodegenerative diseases are incurable, have no treatments, and may result in the progressive degeneration of nerve cells and neural functions. Among these diseases, Alzheimer’s disease (AD) is an age-related neurodegenerative disorder and is a growing problem in developed countries [1,2]. Over the past several decades, AD research has received much funding and attention and has included extensive studies of AD pathogenesis. Much of AD research and many neurologic disease studies have traditionally been based on two-dimensional (2D) cell culture and animal models [3,4]. However, reducing the cost of drug discovery and developing preclinical models that yield results that can be effectively translated into successful therapeutics is urgently needed. It is believed that 2D culture systems fail to construct a brain-like structure and fail to model complex three-dimensional (3D) behavior, such as cell-cell contact and cell-cell interactions. Numerous studies have indicated that the results from cell culture in 3D systems are more physiologically similar to in vivo data and that these 3D systems maintain properties such as viability, differentiation ability, functionality, and protein expression [5,6,7]. Additionally, it is difficult to study cell–molecule interactions and key mechanisms underlying brain disease pathology in animal models, particularly in animal models of neurodegenerative disease. In vitro brain models have the potential to increase our understanding of neural physiology, the potential neurotoxic and biological effects of chemical agents, and the specific cellular mechanisms of neurological disease progression. A brain-on-a-chip would provide an inexpensive and rapid analytical technology to simulate the biophysiological interactions of cells, bridging the gap between conventional 2D culture and animal models for the study of sophisticated brain structures and pharmaceutical applications, such as the screening of new therapeutics and drugs [8].

Technological developments in microfabrication and tissue engineering have led to the progress of organs-on-chips for studying physiology and disease development. These small-scale on-chip models enable the precise control of culture conditions, high-throughput pharmaceutical testing, and basic science development and are promising substitutes for animal testing. Although research on organs-on-chips has gained much attention in the past decade [9], the development of an effective brain-on-a-chip for the study of neurological diseases remains an elusive challenge because of its inherent complexity. In particular, the study of an in vitro brain model should not neglect the simulation of 3D cell-cell interactions and neural networks [10]. Therefore, it is necessary to establish a neurological diseases-on-a-chip model that contains a real-time neural network monitoring system to study the biochemical effects of therapeutic molecules and to examine pathological variations and cell-protein interactions.

The presence of β-amyloid (Aβ) plaques, tau tangles, and oxidative stress are hallmark characteristics of AD. Among these characteristics, the formation of extracellular amyloid plaques consisting of Aβ aggregates is the major pathological hallmark of AD [11,12]. In particular, the Aβ42 variant has been shown to likely be the most toxic for neurons and synapses [13]. Previous reviews have summarized a number of studies about the establishment of neural systems-on-a-chip that enhanced our understanding of the mechanism by which Aβ plaques lead to the loss of synapsis [14], that created an AD model with cocultured healthy and diseased tissues [15], and that modeled the pathophysiological features of AD by mimicking the interstitial space of the brain with a slow flow rate of fluid by an osmotic pump design [16]. A review article also discussed some researches on microfluidic technology in AD biomarker detection and AD physio-pathological processes analysis [17]. Most of these studies focuses on real-time and on-chip visualization to investigate detailed processes of Aβ transmission, aggregation, and neurotoxicity [18,19,20]. Another study also used and analyzed neurofilament dymanics as a fluid biomarker to predict disease progression and brain neurodegeneration at the early presymptomatic stages of familial Alzheimer’s disease [21]. Rankan V.D., et al., have also reviewed the in vivo and in vitro models on Alzheimer’s disease and revealed that the biomimetic model may provide the significant breakthroughs in the areas of AD pathology and therapeutic screening [22]. However, despite the growing appreciation of the need for an in vitro brain model, the neural network formation and neurite degeneration in the AD-on-a-chip model is still hard to monitor and cannot be examined in real time. Especially, a system combining real time investigation and brain-like structure is urgent. S1 prepared a table to compare the advantages of the traditional 2D brain on chip model in previous studies, and the AD on chip model in this study. There are two main advantages in this platform in comparison with other systems. Firstly, the traditional 2D model neglects 3D cell–cell interactions, neural networks formation, controlling the loss of important information to simulate the biochemical effects of therapeutic molecules and to examine pathological variations and cell–protein interactions. Secondly, several systems have tried to construct a 3D model, however the neurite outgrowth direction cannot be controlled, and the neurite connection and disconnection cannot be monitored continuously. Our system provides a real time monitoring system that could be used for tracking the effect of pathological factors and drug screening with time. Herein, a multilayered neural network representing progress towards a brain-on-a-chip was constructed on a micropatterned and material-regulated biochip, building on our previous study [23]. Neural stem cells (NSCs) are the best option for generating a complex and biomimetic environment to model the specific architecture and function of the brain microenvironment. Neural spheroid differentiation and network formation maps created a 3D culture niche, similar to a brain-on-a-chip. Then, the concentration of Aβ was pretested and added into the system to establish AD-on-a-chip. The toxic effects, synapse degeneration, reactive oxygen species (ROS), and neurotransmitter-acetylcholine (ACh) concentrations were examined. In particular, real-time electric cell-substrate impedance sensing records of the network formation and neurite degeneration over three consecutive days were compared with biological data.

## 2. Materials and Methods

### 2.1. Isolation of Cortical NSCs

NSCs were isolated and purified from the cerebral cortex of embryonic day (ED) 14~15 Wistar rat embryos, as described in our previous study with modifications [24]. Animal studies were performed in accordance with the recommendations of the Institutional Animal Care and Use Committee at National Tsing Hua University (IACUC Approval No. 109042, 26 August 2020 approved) and Chang Gung University (IACUC Approval No. CGU106-135, 30 March 2018 approved). Rat embryonic cerebral cortices were isolated, cut into small pieces and mechanically ground in cold Hank’s balanced salt solution (HBSS) on ice. Then, cells were collected by centrifugation and re-suspended in fresh serum-free medium. The cells were counted, and cells were cultured in T25 culture flasks (Corning, NY, USA) at a density of 50,000 cells/cm^2^ in DMEM/F12 medium with bFGF (20 ng/mL, Invitrogen, Waltham, MA, USA) and were maintained in a normal culture environment. Before NSCs spheroid seeding onto the chip, the subculture procedure was repeated to obtain purified NSCs. The images of NSCs spheroid and the immunostaining of nestin for NSC identification before seeding are as shown in S6.

### 2.2. Biochip Fabrication, Neural Network Formation, and AD-on-a-Chip Establishment

**Figure 1:**
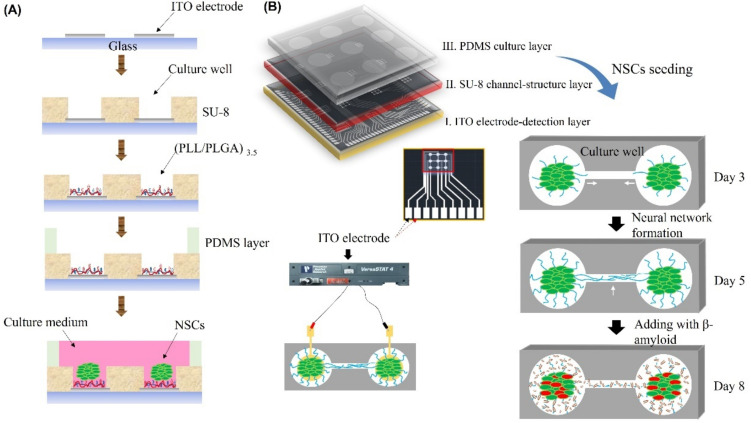
Schematic illustration of the design and fabrication of the AD-on-a-chip model. (**A**) Illustration of the chip fabrication process and NSC seeding. (**B**) Schematic illustration of the 3 layers of the chip, network formation, Aβ addition, network disconnection, and impedance value monitoring. White arrow indicated the neurite outgrowth and neurite connection.

The biochip fabrication and neural network formation was performed as described in our previous study with modifications, as shown in Figure 1A [23]. Briefly, the biochip was constructed with a polydimethylsiloxane (PDMS; Sylgard^®^ 184, Dow Corning, Midland, TX, USA) culture chamber layer, an SU-8 structural layer, and an indium tin oxide (ITO)-glass detector layer, as shown in Figure 1A. In addition, Figure 1B shows three layers of the chip, including the detection layer, SU-8 structure layer, and culture chamber. The SU8 thickness variation versus spin coating speed increase and 3 × 3 array image were as shown in S2. An ITO electrode was embedded in the bottom layer of each culture well. The second layer, the SU-8 structural layer (SU-82050, MicroChem, Round Rock, TX, USA), consisted of a pattern of 3 × 3 arrays of culture wells that were 500 μm in diameter and were connected with channels that were 400 μm long. Then, the structural layer was treated with oxygen plasma and polyelectrolyte multilayer films (PEMs) to guide NSC differentiation. The surface of the pattern layer was modified with (PLL/PLGA)_3.5_ to guide neural network formation, as described in our previous study [23]. As shown in S3, it the mean process length of NSCs on (PLL/PLGA)_3.5_ was about 400 μm after 3 days of culture. Therefore, it is considered that neural network connection on chip could be reached completely after 5 days of culture. The structural layer provides space, holds the neurospheroids in the culture wells, and guides the neurite outgrowth in the channels to ensure complete neural network formation after 5 days of culture. Then, the PDMS provides a chamber layer for culture.

Subsequently, suspension neurospheroids in DMEM/F12 with 0.5% B27 supplement (Gibco, Waltham, MA, USA) were seeded onto the chip, placed into the holes with tweezers and incubated in the same culture environment. After 5 days of culture, 100% of the 3 × 3 neural networks had successfully formed, generating a brain-on-a-chip, as shown in Figure 1B.

Finally, Aβ was added to the brain-on-a-chip system to generate an AD-on-a-chip model, and the biological data were compared with the control group that was not incubated with Aβ. To pretest the Aβ treatment, neurospheroids were incubated with medium containing 1 μM, 3 μM, and 5 μM synthetic Aβ (1–42) (Sigma, San Jose, CA, USA) for 3 days.

### 2.3. Cell Viability and Cytotoxicity Assay (WST1 and Lactate Dehydrogenase (LDH) Assay)

Cell viability was determined by using the cell proliferation reagent WST-1 (ROCHE, Penzberg, Germany) in a colorimetric assay. The WST-1 reagent was directly added to the media and was incubated for 4 h. After the reaction was complete, the plate was then immediately read at 450 nm. A reference reading was performed at 630 nm.

A cytotoxicity Detection Kit (LDH assay) (ROCHE, Penzberg, Germany) was used to measure the LDH released into the culture medium upon cell death due to damage to the plasma membrane. The culture media was directly collected from NSCs incubated with and without different concentrations of Aβ. The LDH content was assessed by an enzyme-linked immunosorbent assay (ELISA) and was read at an absorbance of 490 nm in a multimode microplate reader (BioTek Instruments, Winooski, VT, USA) with a reference wavelength of 630 nm.

### 2.4. Live/Dead Assay

NSCs incubated with/without Aβ were analyzed by using a LIVE/DEAD kit (Life, Orlando, FL, USA). After 3 days of incubation, 100 μL of the dye reagent was mixed with 100 μL of the medium, added to the chip, and incubated at 37 °C for 15 min. After 15 min, photographs of live (green) and dead (red) cells were captured using a digital camera on a fluorescence microscope (Leica, Wetzlar, Germany). The fluorescence intensity of six punched holes was quantified and compared.

### 2.5. Scanning Electron Microscopy (SEM)

SEM (S-300N, Hitachi, Chiyoda, Japan) was used to investigate the morphology of NSCs incubated with different concentrations of Aβ for 3 days. Cells were fixed in ice-cold 2.5% glutaraldehyde in PBS for 1 h and were rinsed three times in PBS for 10 min. Afterward, OsO_4_ was used to fix the cells for 1 h, and the cells were rinsed three times in PBS for 10 min. Then, cells were dehydrated using graded ethanol washes, critical point dried, gold splattered in a vacuum, and examined using SEM.

### 2.6. Immunocytochemistry

Immunocytochemistry detects specific antigens in preserved cell populations by labeling with an antibody. After 8 days of incubation, cells were fixed in 70% methanol for 5 min and were rinsed three times with PBS. After fixing, primary monoclonal antibodies were diluted to an appropriate concentration in a solution containing 0.3% Triton X-100 and 10% bovine serum albumin (BSA). Then, the cells were incubated with the following primary antibodies for 2 h at 37 °C: an anti-microtubule associated protein 2 (MAP-2) monoclonal antibody (1:500; Millipore, Burlington, MA, USA) was utilized to identify neurons, and anti-Synapsin I (1:500; Millipore, Germany) was utilized to identify neurites. The cells were then incubated with the following secondary antibodies for 30 min at room temperature: FITC- and rhodamine-conjugated secondary antibodies (1:250, AP187F; AP181 R; Millipore, Germany) and an anti-Hoechst 33,342 monoclonal antibody (1:2000; Cat. no.: H3570; Invitrogen, Waltham, MA, USA). In addition, amyloid plaque staining was performed after fixation. After fixation, NSCs were washed with PBS three times and were incubated in 1 μM Thioflavin S (Sigma Aldrich, Schnelldorf, Germany), which was diluted in 50% ethanol, for 10 min. Then, the samples were rinsed with 80% ethanol twice for 5 min and were washed with PBS three times for 3 min. A drop of fluorescent mounting medium (Dako, Carpinteria, CA, USA) was added to each slide, and slides were sealed with a coverslip. Finally, samples were visualized by indirect fluorescence under a confocal fluorescence microscope (LSM 510 META; Zeiss, Jena, Germany).

### 2.7. ROS Detection

An ROS assay kit (BioVision, Milpitas, CA, USA) was used to detect the accumulation of intracellular ROS in NSCs after 24 h of incubation with Aβ. NSCs were cultured on a chip for network formation and were subsequently incubated with/without 5 μM Aβ for 24 h. After removing the medium, 100 μL of 2′,7′-dichlorofluorescin diacetate (DCFDA) solution was added to each well and incubated for 45 min at 37 °C in the dark. The fluorescence intensity was quantified by using ImageJ with a 3 × 3 array.

### 2.8. Impedance Measurement of the Neural Network Connections

The electrical connection of each pair of neurospheroids could be determined by measuring the impedance across each pair of electrodes. The impedance measurement was conducted by using an impedance analyzer (VersaSTAT 4; Princeton Applied Research, Oak Ridge, TN, USA). A potential of 0.1 Vrms was applied across the electrodes, and the impedance data from 1 Hz to 10 kHz were collected for each measurement. Based on our previous study, impedance at 1 kHz and a threshold of 40 kΩ was used to determine the electrical connections of neurospheroids [23].

### 2.9. ACh Assay

The ACh concentration of NSCs after 5 days of culture with/without 3 days of Aβ incubation was measured by using a colorimetric assay kit (Bioassay Systems, Hayward, CA, USA) according to the manufacturer’s instructions. First, lysis buffer was added to the chip and incubated for 15 min at room temperature (RT). Then, 20 µL of sample was mixed with 80 µL of working reagent and was incubated in the dark for 30 min. The optical density (OD) of the samples was recorded at 570 nm. The color intensity at 570 nm was directly proportional to the ACh concentration in the sample and was calculated from the standard curve.

### 2.10. Statistical Analysis

All of the quantification results are expressed as the mean values ± the standard deviation (SD) from each independent experiment. Sigma Plot version 14.0 (Systat Software, Inc.) was used for analyses. Each experiment was repeated 3 times. A Student’s *t*-test was used to evaluate the statistical significance and is indicated as follows: * *p* < 0.05, ** *p* < 0.01, *** *p* < 0.005, and **** *p* < 0.001.

## 3. Results and Discussion

### 3.1. Effect of the Aβ Concentration on NSCs

The effect of the Aβ concentration on neurospheroids on a biochip was pretested, and the results of cell viability assay and SEM photograph of NSCs incubated with 1 μM, 3 μM, and 5 μM of Aβ for three days are shown in Figure S4. As shown in Figure S4A, cell viability significantly decreased after NSCs were incubated with 1 μM, 3 μM and 5 μM of Aβ for three days. The higher Aβ concentration group displayed a lower cell viability. However, there was no significant difference in the OD value between the groups incubated with 3 μM Aβ and 5 μM Aβ. Moreover, the SEM photographs of NSCs morphologies after incubation with different concentrations of Aβ are shown in Figure S4B. As shown in Figure S4, cell attachment, migration from the spheroid, and process outgrowth were observed in the group without Aβ incubation. In contrast, varying degrees of cell death and neurite degeneration were observed in the three groups with Aβ incubation, includes1 μM, 3 μM and 5 μM of Aβ incubation. In particular, numerous shriveled cells and neurite damage were observed in the group incubated with 5 μM Aβ, as shown in Figure S4. Therefore, a concentration of 5 μM Aβ was considered suitable for use in this system to model neuronal damage and network degeneration in AD.

### 3.2. The Addition of Aβ to the Biochip to Generate an In Vitro AD Model

**Figure 2:**
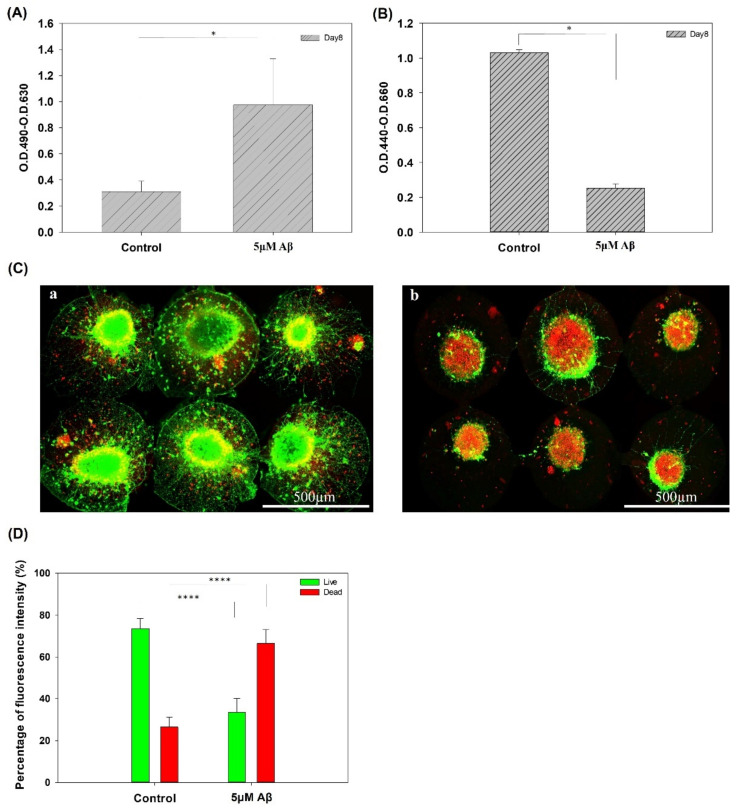
(**A**) LDH assay of NSCs on a chip after 5 days of network formation and incubated with/without 5 μM Aβ for 3 days. (**B**) Cell viability assay of cells on a chip after 5 days of network formation incubated with/without 5 μM Aβ for 3 days. (**C**) Live/dead staining of NSCs on a chip after 5 days of network formation and incubated (**a**) with/(**b**) without 5 μM Aβ for 3 days. (**D**) Quantification of the percentages of live and dead cells (* *p* < 0.05, **** *p* < 0.001).

Figure 2A,B show the cytotoxicity and cell viability of NSCs with/without Aβ after 3 days of incubation, respectively. Both the LDH assay and WST1 assay showed a significant difference between the control and Aβ treatment groups. In addition, the results of the live/dead staining in the groups with/without incubation with 5 μM Aβ are shown in Figure 2C,D, respectively. The quantification results of the live/dead staining are shown in Figure 2D. In the control group, after eight days of culture, most of the NSCs were alive, and few dead cells were observed. The nutrients and medium in the chip are limited, resulting in a small amount of cell death. In contrast, few live cells and a large number of dead cells were observed in the chip with 5 μM Aβ, and the results were significantly different in the control and 5 μM Aβ groups.

**Figure 3:**
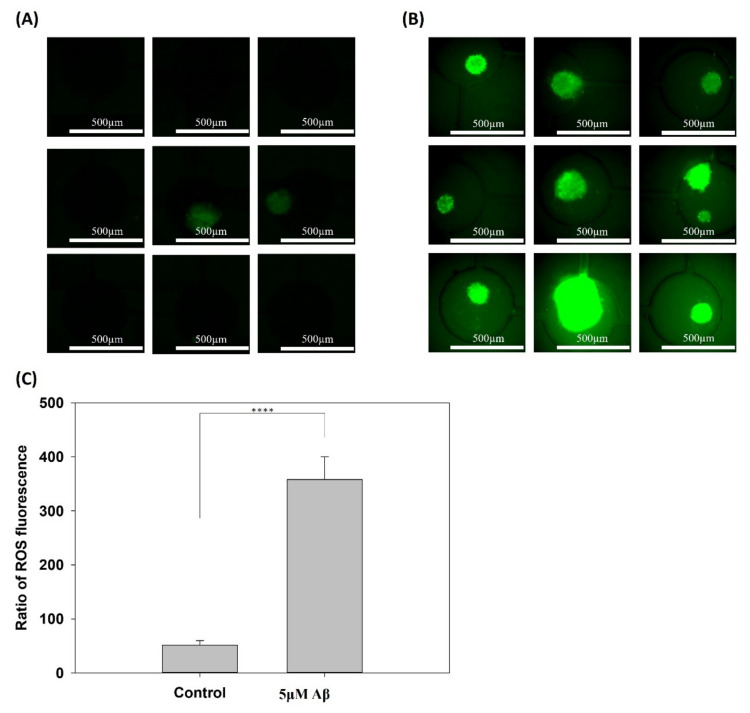
Production of ROS in NSCs cultured on a chip after 5 days of network formation and incubated with/without 5 μM Aβ for 3 days. (**A**) Control group incubated without 5 μM Aβ and (**B**) with 5 μM Aβ for 3 days. (**C**) Quantification of the fluorescence intensity of ROS production (**** *p* < 0.001).

In addition, ROS are toxic agents involved in several neurodegenerative diseases, including AD and brain dysfunction due to injury or aging. Several studies have shown evidence indicating that the aggregation of Aβ and tau is a compensatory response to underlying oxidative stress [25,26]. Therefore, the accumulation of ROS in the control and 5 μM Aβ treatment groups was determined and is shown in Figure 3A,B, respectively. The ROS fluorescence of the 3 × 3 array in the control and 5μM Aβ treatment groups was quantified, and the fluorescence intensity was normalized to the sphere area, as shown in Figure 3C. Figure 3A shows that there was little ROS release in the control group and a very high fluorescence intensity in all of the 3 × 3 arrays in the 5 μM Aβ treatment group, which suggested that a great quantity of ROS was released only one day after Aβ was added. Figure 3C shows the quantification results and that the ROS in the 5 μM Aβ treatment group was significantly different (*p* < 0.001) than that in the control group.

**Figure 4:**
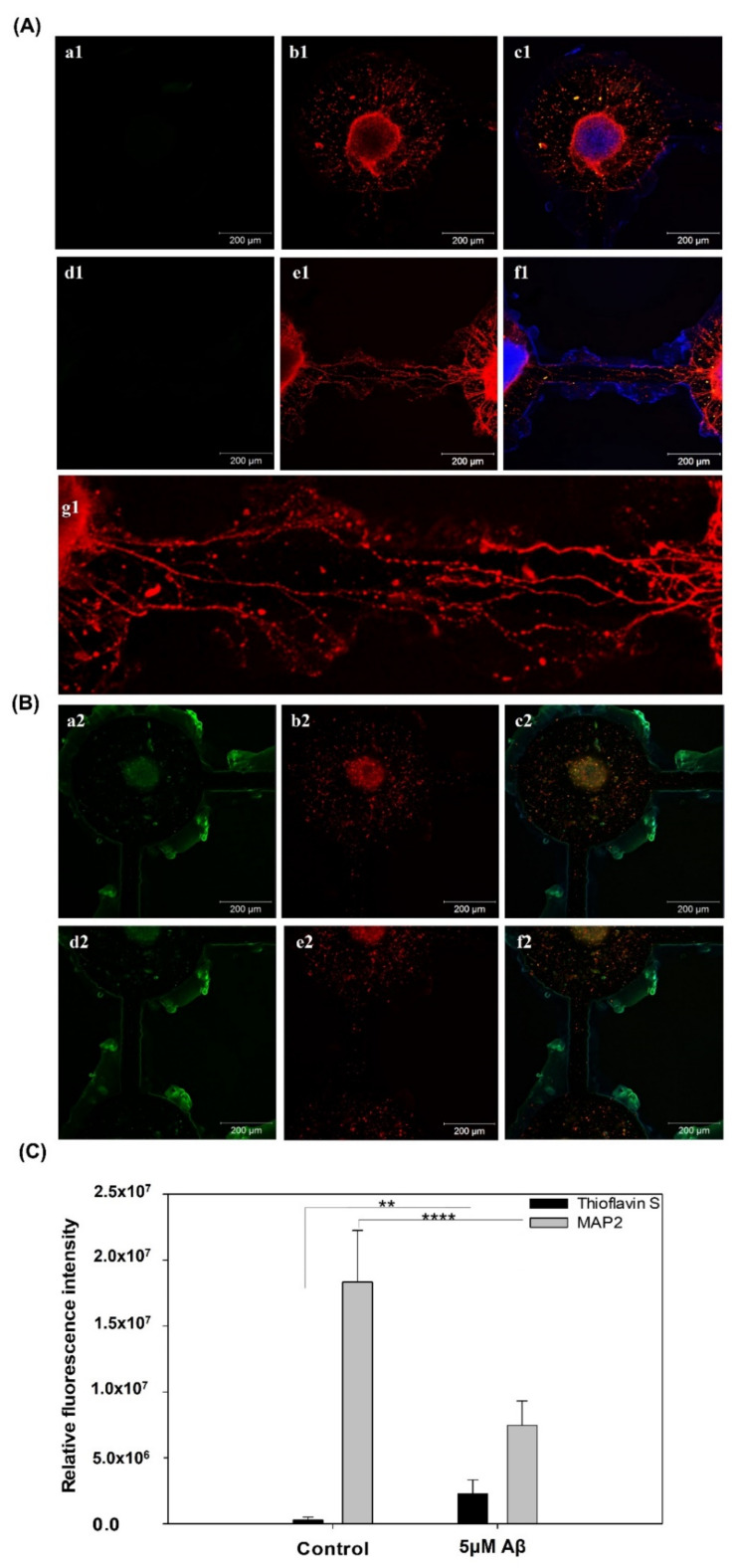
Immunofluorescence staining of Thioflavin S protein (green), MAP2 (red), and Hoechst 33342 (blue) expression in NSCs cultured on a chip after 5 days of network formation and incubated (**A**) without (**B**) with 5 μM Aβ for 3 days. (**C**) Quantification of the fluorescence intensity of Thioflavin S protein and MAP2 expression (** *p* < 0.01 and **** *p* < 0.001).

Furthermore, the immunostaining results of MAP2 and Thioflavin S are shown in Figure 4, showing the neuron percentage and the quantification of Aβ aggregation. The MAP2 (red) and Thioflavin S (green) staining in the control and 5 μM Aβ treatment groups are shown in Figure 4A,B, respectively. As shown in Figure 4A(b1), neuron cell differentiation and neurite outgrowth were clearly observed. In addition, the neurite outgrowth in the channels connecting two neurospheroids is shown in Figure 4A(e1) and the magnification images of the connection is shown in Figure 4A(g1). In contrast, as shown in Figure 4B(b2,e2), a large number of neurites were damaged, and the connections between neurites in the channel were clearly disrupted. The neural network was maintained after eight days of culture in the brain-on-a-chip system, and the neurites were highly degenerated in the AD-on-a-chip group. Moreover, the Thioflavin S staining displays a redshift in the emission spectrum when it binds to beta sheet-rich structures, such as amyloid aggregates [27]. Figure 4B also shows that a large amount of Aβ accumulated in the AD-on-a-chip group compared to the control group. As shown in Figure 4C, the neuron expression significantly decreased and Aβ aggregation significantly increased in the 5 μM Aβ treatment group. These results showed that Aβ aggregation resulted in neuronal damage and neurite degeneration.

### 3.3. Real-Time Impedance Analysis Monitors Network Connection and Disconnection

**Figure 5:**
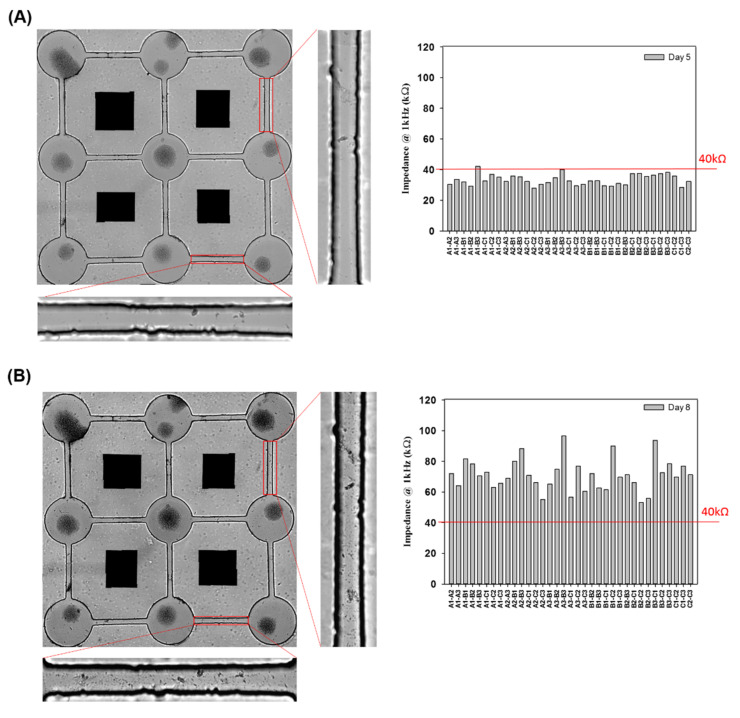
Impedance analysis of the neural network formation and disconnection. (**A**) Images of NSCs on a biochip after 5 days of culture with a 100%-connected 3 × 3 neural network and all the impedance values between each pair of electrodes. (**B**) Images of a disconnected neural network incubated with 5 μM Aβ for 3 days and the impedance values between each pair of electrodes.

**Figure 6:**
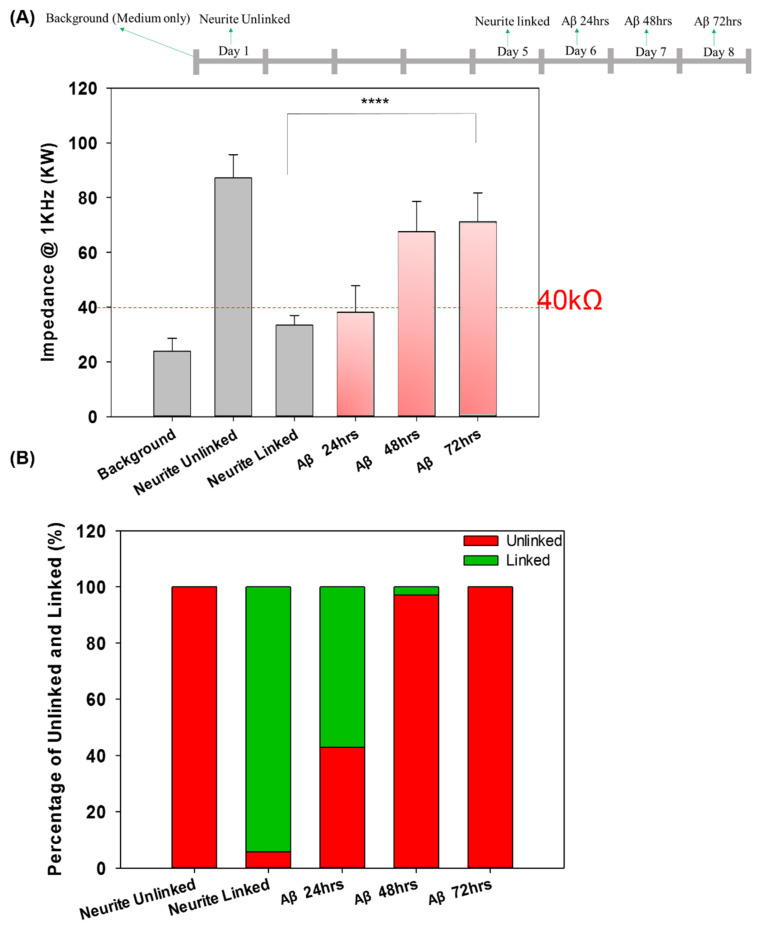
(**A**) The impedance values determined for each pair of electrodes for the background (medium only), unconnected neurites, connected neurites, and the system incubated with 5 μM Aβ for 24 h, 48 h, and 72 h. (**B**) The percentage of unlinked and linked neurites in the control group and in the system incubated with 5 μM Aβ for 24, 48 and 72 h (**** *p* < 0.001).

Our previous study investigated the neural network connection by microscopic imaging and compared the impedance values as a proof-of-concept for the impedance analysis [23]. Herein, the network connection and disconnection were continuously monitored, and the impedance of each pair of neurospheroids was recorded. We compared the large number of impedance values for the background (medium only), unconnected neurites in each pair of neurospheroids, and connected neurites in each pair of neurospheroids. A threshold of 40 kΩ was defined for the determination of the electrical connection of neurospheroids. When the neurites connected two neurospheroids, the impedance value was reduced as the electrical connection was generated. In contrast, in the AD model, when the neurites were damaged by Aβ and were disconnected, the effective electrode surface area was reduced, which led to an increase in the impedance values. To confirm the impedance value effect of Aβ accumulation, the impedance value of 5 μM Aβ in medium without NSCs was determined and is shown in Figure S5. As shown in Figure S5A, cell morphology with Aβ accumulation was observed after 1 h, one day, two days, and three days of incubation. In addition, Figure S5B shows that the impedance variation caused by Aβ accumulation after one day, two days, and three days of incubation was very small and was not significantly different than the value of the medium alone. Figure 5A showed a complete 3 × 3 neural network after five days of culture, and the impedance values between every pair of electrodes in the 3 × 3 neural network were all below 40 kΩ, which was consistent with our previous study [23]. These data confirmed the electrical connection between each pair of neurospheroids in the 3 × 3 neural network. Figure 5B shows that after three days of incubation with 5 μM Aβ, all of the impedance values in the 3 × 3 neural network increased (over 40 kΩ), and the images also show neurite disconnection, which was consistent with the impedance analysis. Furthermore, the impedance analysis could be used to consecutively investigate the neurite connection and disconnection in real time without cumbersome image taking. As shown in Figure 6A, the average impedance values in the 3 × 3 neural network were all recorded, including the values of the medium alone, after one day of culture (unlinked neurites) and five days of culture (linked neurites), after 24 h of incubation with 5 μM Aβ, after 48 h of incubation with 5 μM Aβ, and after 72 h of incubation with 5 μM Aβ. The impedance values in AD model in detail were as shown in Figure S7. After one day of culture, all of the impedance values in the 3 × 3 neural network were above 40 kΩ, and the average impedance value was approximately 87.18 kΩ. After five days of culture, 97% of the recorded impedance values in the 3 × 3 neural network were below 40 kΩ, and the average value was approximately 33.4 kΩ. In the AD model, it was demonstrated that after incubation with 5 μM Aβ, the average impedance value increased as the incubation time increased. Figure 6B shows the relative percentages of linked neurites and unlinked neurites, using 40 kΩ as the threshold. After incubation with 5 μM Aβ, the neurite disconnection percentage increased with the incubation time, with an unlinked percentage of over 40% after 24 h and up to 100% after 72 h of incubation. The impedance analysis performed in this study was consistent with microscopic imaging analysis. This system demonstrated a method to monitor neural network connections/disconnections in real time, allowing the convenient, consecutive, and quantitative investigation of AD on a chip and providing an alternative to in vitro models of different neurological diseases.

### 3.4. Synapse Function Determination and ACh Determination

**Figure 7:**
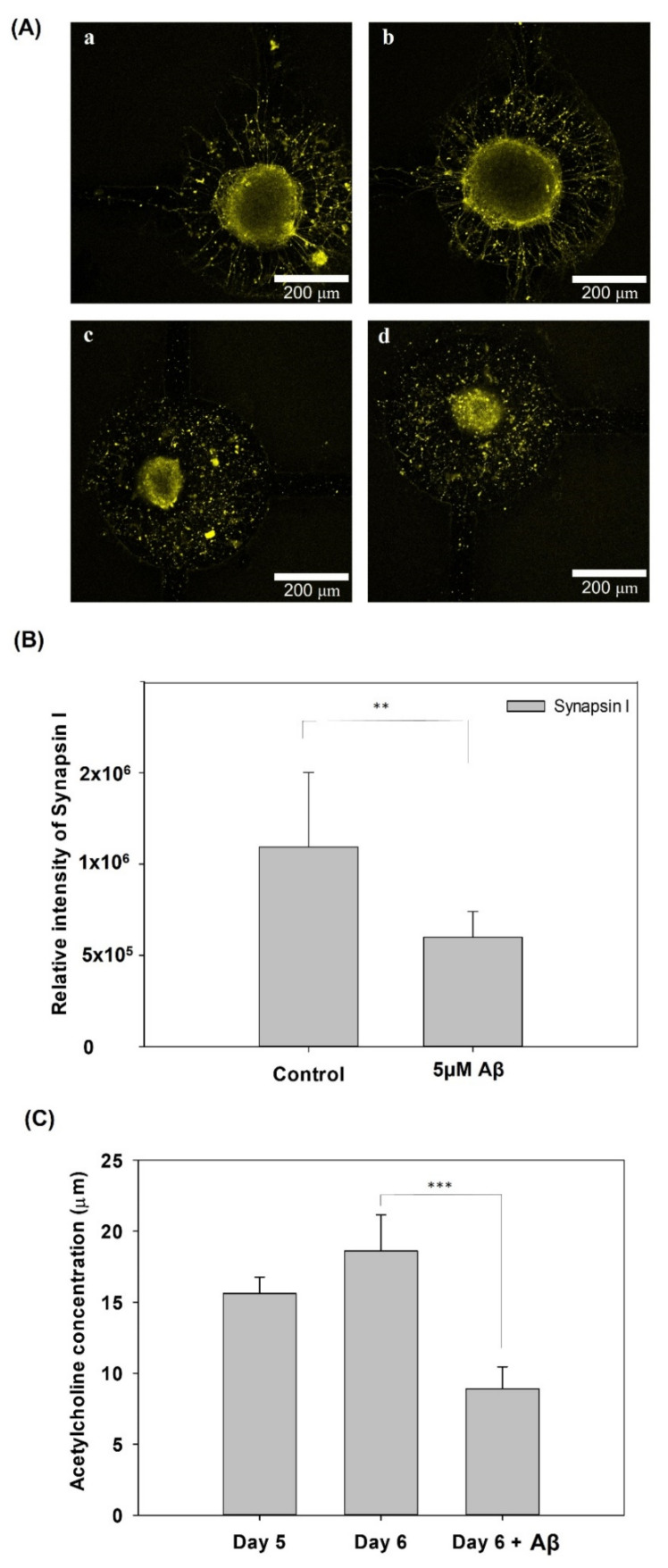
(**A**) Immunofluorescence staining of synapsin I expression in NSCs on a biochip after 5 days of culture and incubated with (**c**,**d**)/without (**a**,**b**) 5 μM Aβ for 3 days. (**B**) Quantification of the relative fluorescence intensity of synapsin I. (**C**) Analysis of ACh release from NSCs on a biochip after 5 days of network formation and incubated with or without 5 μM Aβ for 1 day (** *p* < 0.01 and *** *p* < 0.005).

To further confirm the damage to neurite function in the AD model, Figure 7 shows the synapse immunostaining results and the ACh concentration after five days of culture and on day 6 with/without incubation with 5 μM Aβ. Figure 7A shows that the fluorescence of synapsin I in the control group was higher than that in the group with 5 μM Aβ. In addition, as shown in Figure 7(Aa,Ab), the neurites around the neurospheroids was thick, dense and very clear. In contrast, as shown in Figure 7(Ac,Ad), the neurites around the neurospheroids were disconnected and damaged. Furthermore, the quantification of the relative fluorescence intensity was also significantly different between the control and the 5 μM Aβ treatment groups. Furthermore, ACh is a kind of neurotransmitter released by nerve cells and functions in the brain at the neuromuscular junction. At the cellular and synaptic levels, ACh causes a variety of responses depending on the specific ACh receptors present on the cell [28]. In addition, ACh plays a crucial role in the central nervous system and is involved in learning, memory, and movement [29]. The dysfunction in ACh regulation in the brain results in neuropsychiatric disorders, including AD. Several previous studies have tried to use ACh as a biomarker for early diagnosis and the evaluation of pharmaceutical treatment effectiveness [30,31]. Figure 7C shows the quantification of ACh concentrations after five and six days of culture with or without Aβ incubation. The ACh concentration was downregulated to 8.92 ±1.55 μM in the group treated with Aβ after only one day of incubation, which represented a significant decrease compared with the group without Aβ incubation (*p* < 0.005). These results demonstrated that incubation with 5 μM Aβ in a neural network system not only induced amyloid accumulation, neural network atrophy, and ROS increases, but also resulted in synapse damage and dysfunction, which is consistent with the symptoms of AD; thus, this system successfully modeled AD in vitro.

## 4. Conclusions

An NSC-based neural network representing progress towards a brain-on-a-chip was successfully constructed, and the incubation of the chip with Aβ generated an AD-on-a-chip model. NSC spheroids generated a brain-like structure with network formation, generating a highly accurate brain model. This platform generated a biomimetic in vitro AD model that allowed the unique, real-time monitoring of network connection/disconnection. This model has great potential for drug discovery and the development of novel therapeutic methods and could also be used to establish and modulate other in vitro neurological disease models.

## Data Availability

Not applicable.

## Supplementary Materials

The following supporting information can be downloaded at: https://www.mdpi.com/article/10.3390/pharmaceutics14051022/s1, Figure S1: In comparison of the model developed in this study with the traditional 2D model, and other brain models; Figure S2: (a) The SU8 thickness variation versus spin coating speed increase. (b) Image of a 3 × 3 array; Figure S3: Quantification of the lengths of the processe length of NSCs on the PLL/PLGA multilayer films under serum-free conditions after 3 days of culture. The lengths of the 10−15 longest processes per neurosphere were estimated linearly from the edge of the neurospheres to the tip of the processes; Figure S4: (A) Cell viability assay of NSCs incubated with 1 μM, 3 μM and 5 μM of Aβ for 3 days. (B) Morphologies of NSCs incubated with (a) 0, (b) 1 μM, (c) 3 μM and (d) 5 μM of Aβ for 3 days; Figure S5: (A) The morphology of cells in the system after incubation with Aβ for (a) 1 h (b) 1 day (c) 2 days and (c) 3 days at 37 °C with 5% CO_2_. (B) The variation in the impedance value in response to incubation with Aβ for 1, 3, and 5 days at 37 °C with 5% CO_2_; Figure S6: The images of NSCs spheroid and the immunostaining of nestin for NSC identification. (A) Phase image (B) Nestin expression; Figure S7: All the impedance values between each pair of electrodes on a biochip after 5 days of culture (day 5) and incubated with 5 μM Aβ for 3 days (day 6, day 7 and day 8).
