# Supplementary material for: A Real-Time Sensing System for Monitoring Neural Network Degeneration in an Alzheimer’s Disease-on-a-Chip Model
Source: Pharmaceutics. 2022 May 9;14(5):1022. doi: 10.3390/pharmaceutics14051022 (PMC9148060; doi:10.3390/pharmaceutics14051022)
Supplement: Supplementary file 1 [file pharmaceutics-14-01022-s001.zip › pharmaceutics-1695819-supplementary.pdf]

A Real-Time Sensing System for Monitoring Neural Network Degeneration in an Alzheimer's Disease-on-a-Chip Model

Nien-Che Liu [1], Chu-Chun Liang [1], Yi-Chen Ethan Li [2] and I-Chi Lee [1, *]

[1] Department of Biomedical Engineering and Environmental Sciences, National Tsing Hua University, Hsinchu, 300044, Taiwan

[2] Department of Chemical Engineering, Feng Chia University, Taichung, 40724, Taiwan

* **Corresponding author: I-Chi Lee**

Mailing address: 300044 No. 101, Section 2, Kuang-Fu Road, Hsinchu, Taiwan, R.O.C.

E-mail: iclee@mx.nthu.edu.tw

System / Advantages	Our brain model	Traditional 2D brain model	Other brain model	
Culture system	2.5D	2D	2.5D	3D
Biomimetic/dynamic system	Δ/X	X/Δ	Δ/Δ	Δ/Δ
Channels connect adjacent spheres	✓	X/Δ	X/Δ	X
To control and monitor neurite connection	✓	X	X/Δ	X
Real-time monitoring	✓	X	X/✓	X/Δ

Figure S1. In comparison of the model developed in this study with the traditional 2D model, and other brain models.

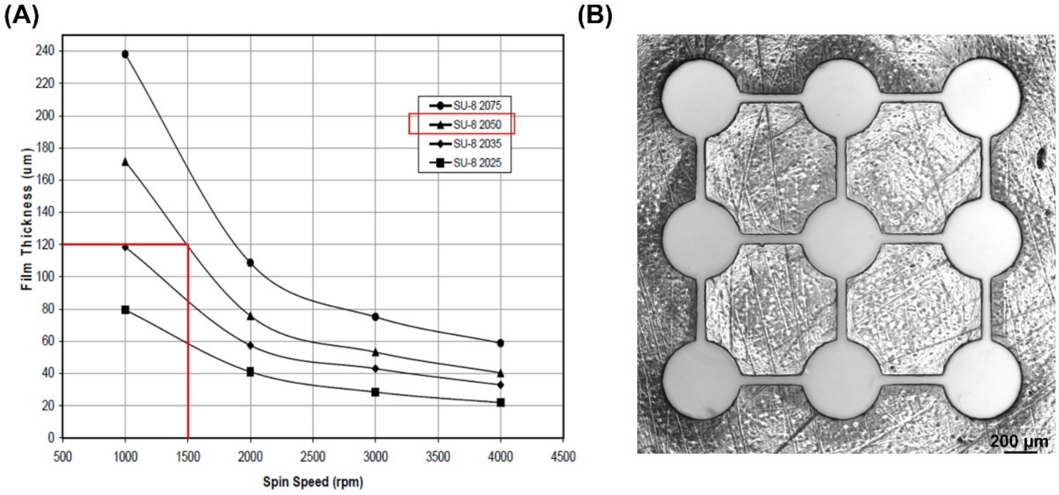

Figure S2. (a)The SU8 thickness variation versus spin coating speed increase. (b) Image of a 3x3 array.

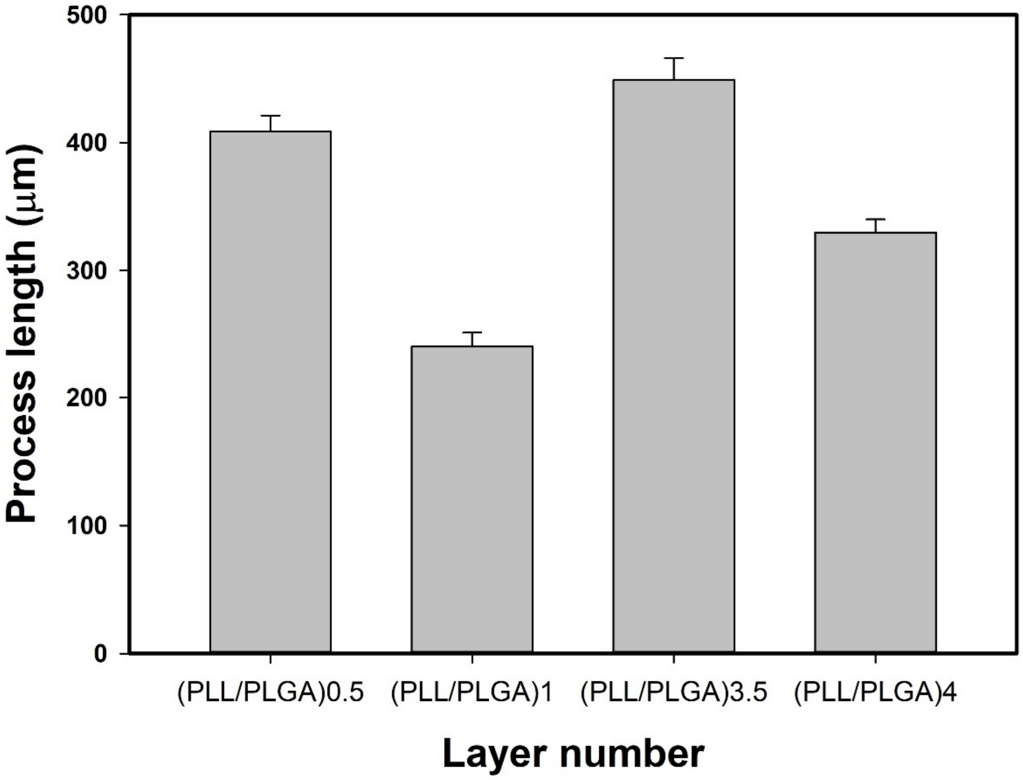

Figure S3. Quantification of the lengths of the processe length of NSCs on the PLL/PLGA multilayer films under serum-free conditions after 3 days of culture. The lengths of the 10−15 longest processes per neurosphere were estimated linearly from the edge of the neurospheres to the tip of the processes.

(A)

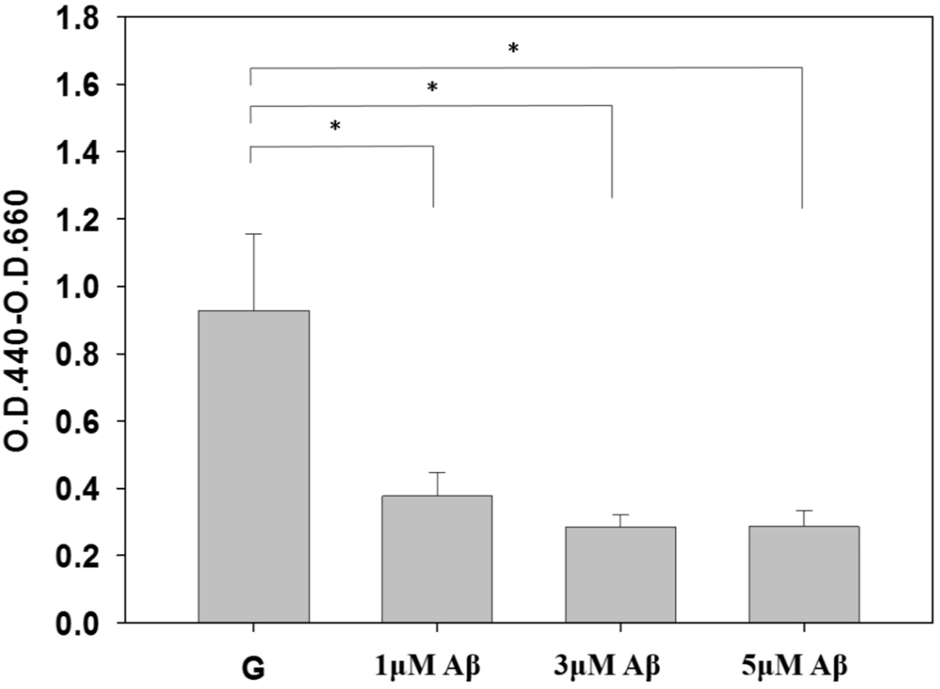

(B)

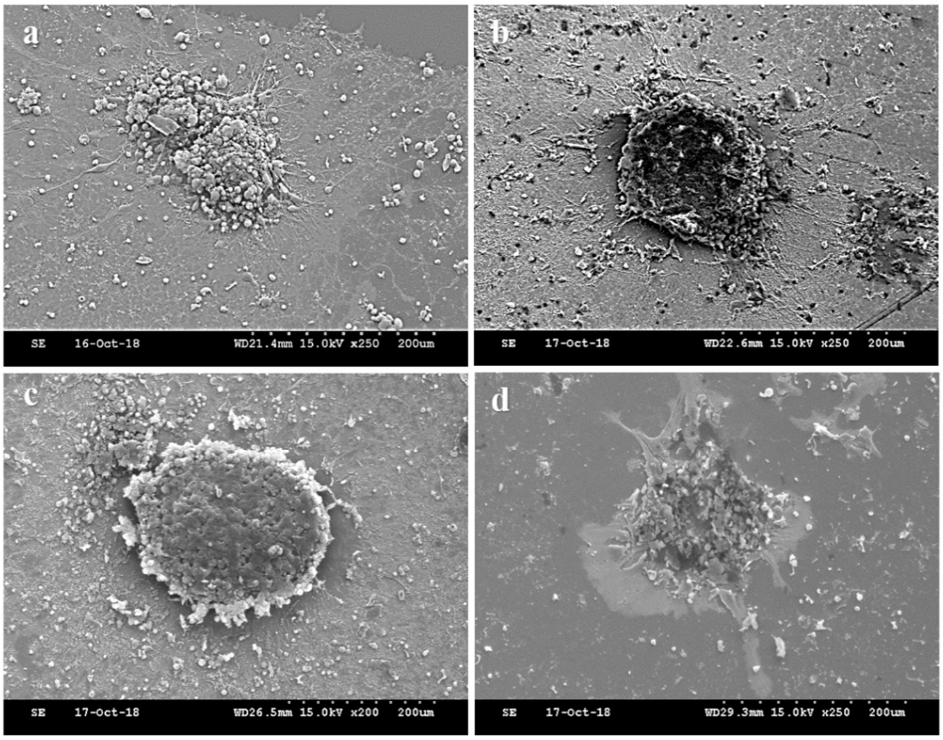

Figure S4. (A) Cell viability assay of NSCs incubated with 1 μM, 3 μM and 5 μM of Aβ for 3 days. (B) Morphologies of NSCs incubated with (a) 0, (b) 1 μM, (c) 3 μM and (d) 5 μM of Aβ for 3 days.

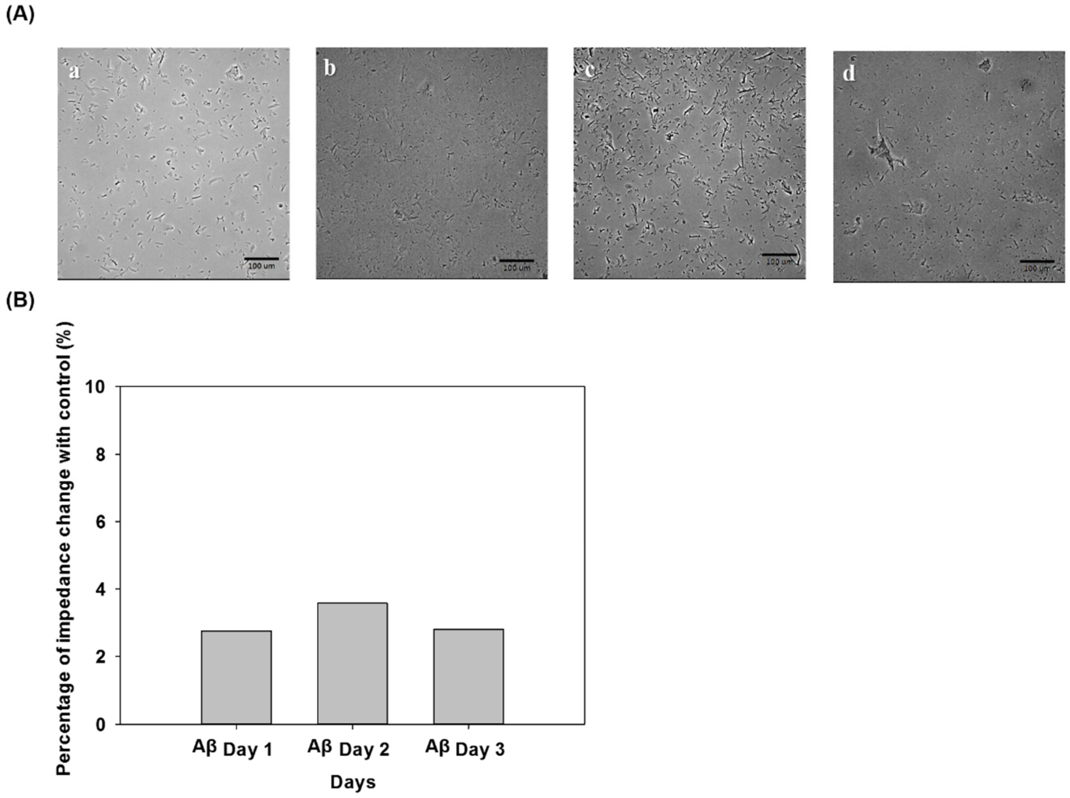

Figure S5. (A) The morphology of cells in the system after incubation with Aβ for (a) 1 hr (b) 1 day (c) 2 days and (c) 3 days at 37 °C with 5% CO_2. (B) The variation in the impedance value in response to incubation with Aβ for 1, 3, and 5 days at 37 °C with 5% CO_2.

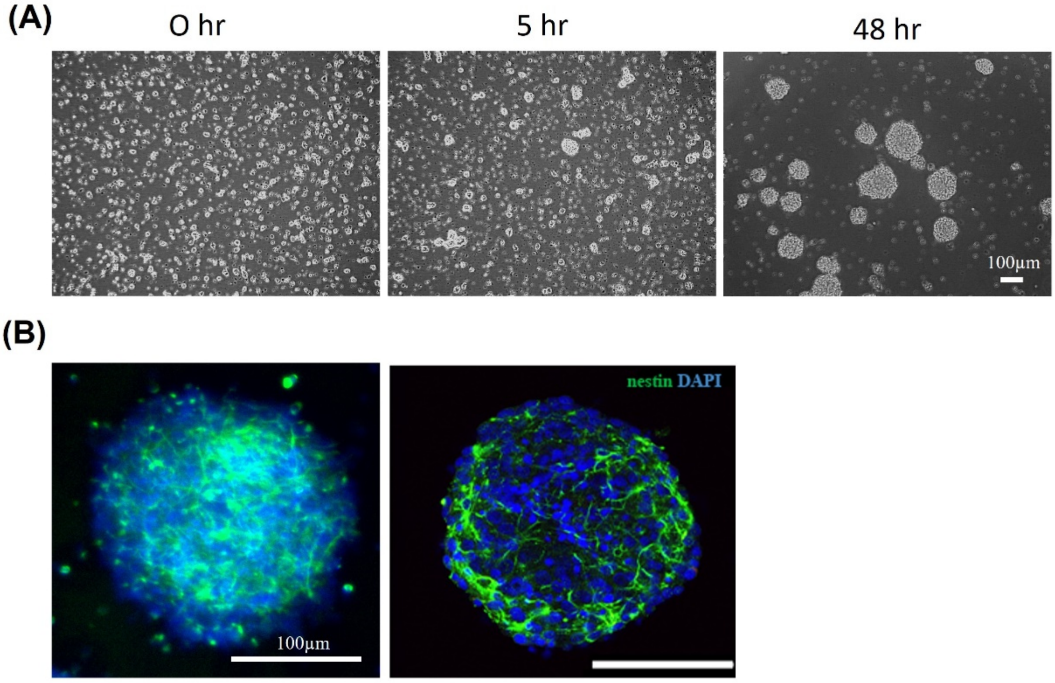

Figure S6. The images of NSCs spheroid and the immunostaining of nestin for NSC identification. (A) Phase image (B) Nestin expression.

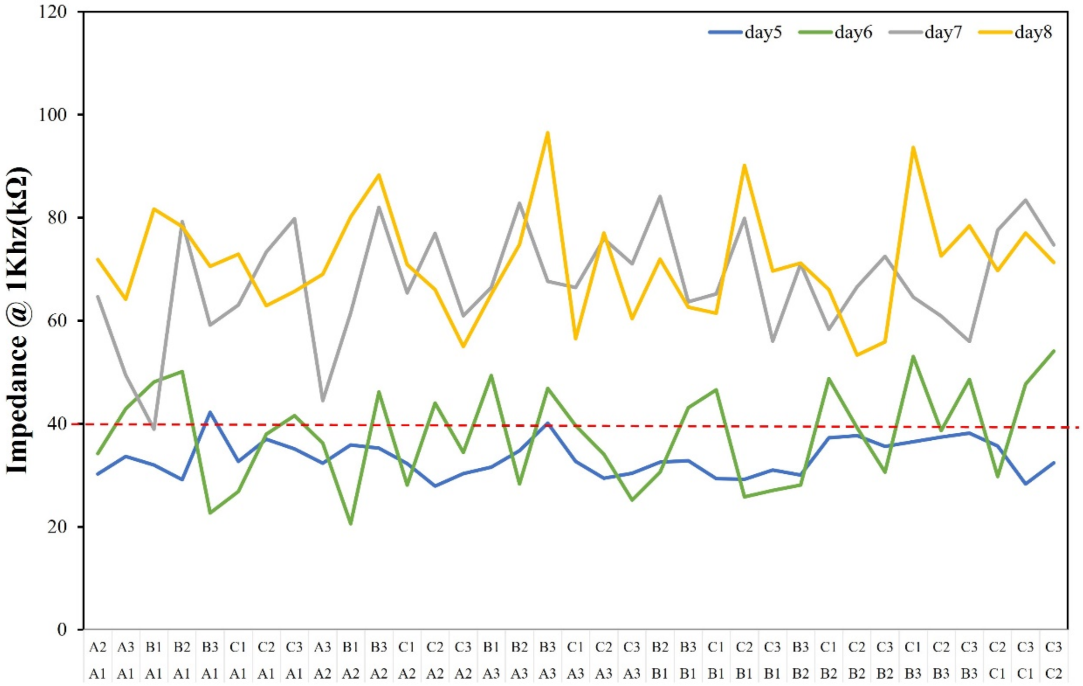

Figure S7. All the impedance values between each pair of electrodes on a biochip after 5 days of culture (day 5) and incubated with 5 μM Aβ for 3 days (day6, day7 and day8).
